# Controversial Effects of Vitamin D and Related Genes on Viral Infections, Pathogenesis, and Treatment Outcomes

**DOI:** 10.3390/nu12040962

**Published:** 2020-03-30

**Authors:** Choongho Lee

**Affiliations:** College of Pharmacy, Dongguk University, Goyang 10326, Korea; choongholee@dongguk.edu; Tel.: +82-31-961-5223

**Keywords:** vitamin D (VD), vitamin D-related genes (VDRG), viral infection, pathogenesis, treatment outcome

## Abstract

Vitamin D (VD) plays an essential role in mineral homeostasis and bone remodeling. A number of different VD-related genes (VDRG) are required for the metabolic activation of VD and the subsequent induction of its target genes. They include a set of genes that encode for VD-binding protein, metabolic enzymes, and the VD receptor. In addition to its well-characterized skeletal function, the immunoregulatory activities of VD and the related polymorphisms of VDRG have been reported and linked to its therapeutic and preventive actions for the control of several viral diseases. However, in regards to their roles in the progression of viral diseases, inconsistent and, in some cases, contradictory results also exist. To resolve this discrepancy, I conducted an extensive literature search by using relevant keywords on the PubMed website. Based on the volume of hit papers related to a certain viral infection, I summarized and compared the effects of VD and VDRG polymorphism on the infection, pathogenesis, and treatment outcomes of clinically important viral diseases. They include viral hepatitis, respiratory viral infections, acquired immunodeficiency syndrome (AIDS), and other viral diseases, which are caused by herpesviruses, dengue virus, rotavirus, and human papillomavirus. This review will provide the most current information on the nutritional and clinical utilization of VD and VDRG in the management of the key viral diseases. This information should be valuable not only to nutritionists but also to clinicians who wish to provide evidence-based recommendations on the use of VD to virally infected patients.

## 1. Vitamin D and Vitamin D-Related Genes

Vitamin D (VD) is an essential micronutrient required for the intestinal absorption of minerals such as calcium, magnesium, and phosphate and the maintenance of bone strength. This fat-soluble secosteroid hormone is a key regulator necessary for overall mineral homeostasis and bone remodeling [1]. The classical calcium-related skeletal functions of VD are well established from a historical perspective due to the clear causal association of VD deficiency with the development of rickets, a form of osteomalacia in young children [2]. Due to the critical role of VD in the prevention of bone-related diseases, VD supplementation has been recommended for populations who are at high risk for the development of VD deficiency. The beneficial effects of VD supplementation on other medical conditions such as cancer, cardiovascular, and infectious diseases have been well documented. However, despite these encouraging results, numerous studies have also reported unclear and sometimes conflicting data on the effects of VD supplementation on the progression or prevention of certain diseases [3,4,5,6]. In particular, with regard to its non-skeletal effects on general mortality, cancer, and infectious diseases, the nutritional and therapeutic benefits of VD supplementation have been controversial and inconclusive [4,7,8].

**Figure 1:**
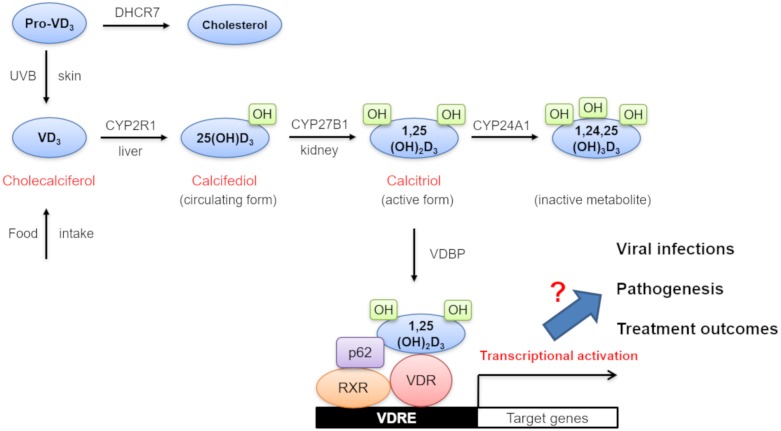
Diagram of vitamin D (VD) synthesis, metabolism, biological action, and its potential effects on viral infections, pathogenesis, and treatment outcomes. Abbreviations: VD_3_, vitamin D_3_; DHCR7, 7-dehydrocholesterol reductase (DHCR7); UVB, ultraviolet B; 25(OH)D_3_, 25-hydroxy vitamin D_3_; 1, 25(OH)D_3_, 1, 25-dihydroxy vitamin D_3_; RXR, retinoic acid receptor; VDR, vitamin D receptor; VDRE, vitamin D responsive elements.

VD_3_ (cholecalciferol) is generated via endogenous UVB-dependent photochemical synthesis from pro-VD_3_ in the skin or through the dietary intake [9,10] (Figure 1). It is then converted into 25-hydroxy VD_3_ (25(OH)D_3_) (calcifediol) by a hepatic VD_3_ 25-hydroxylase (CYP2R1) [10]. 25(OH)D_3_ levels are frequently measured in serum to determine VD status since it is the major circulating form of VD. The second round of 25(OH)D_3_ hydroxylation is further facilitated by a renal 25-hydroxy VD_3_-1α-hydroxylase (CYP27B1) and results in the production of 1,25-dihydroxy VD_3_ (1,25(OH)_2_D_3_) (calcitriol), which is an active form of VD [11]. 1,25(OH)_2_D_3_ is then transported to local tissues with the help of an α-globulin carrier protein called VD-binding protein (VDBP). After its intracellular uptake, 1,25(OH)_2_D_3_ binds to its cognate VD receptor (VDR) in the cytoplasm [12]. This 1,25(OH)_2_D_3_-bound VDR creates a complex with a retinoic acid receptor (RXR) and p62 in the nucleus. Subsequent association of this trimeric 1,25(OH)_2_D_3_-bound VDR/RXR/p62 complex with VD-responsive elements (VDRE) in the promoter regions of target genes drives the transcriptional activation of a set of VD-responsive genes. For the neutralization of VD, 1,25(OH)_2_D_3_ is further converted into an inactive metabolite (1,24,25(OH)_3_D_3_) by another hydroxylase enzyme, CYP24A1. In this paper, all genes involved in VD metabolism, activation, and degradation are collectively designated as VD-related genes (VDRG). As shown in Figure 1, they include 7-dehydrocholesterol reductase (DHCR7), VDBP, CYP2R1, CYP27A1, CYP27B1, CYP24A1, and VDR.

## 2. VD Deficiency and the Controversial Roles of VD in the Development of Viral Diseases

Plasma VD levels are closely linked to changing seasons due to the dependence of VD activation on UV radiation [9,10]. Besides, numerous epidemiological studies have confirmed the impacts of geographical location on plasma VD levels due to sun exposure variation among different latitudes. Under normal conditions, the calcium-parathyroid hormone-VD axis is involved in the maintenance of optimal plasma calcium levels. VDBP also plays an important role in the regulation of serum VD levels through its plasma transportation capability [13]. In 2011, an Institute of Medicine committee concluded that a 25(OH)D_3_ serum level of 20 ng/mL (50 nmol/L) is necessary for overall bone health [14]. Based on this recommendation, VD deficiency is generally defined as a plasma 25(OH)D_3_ level less than 20 ng/mL (50 nmol/L).

As mentioned previously, the cause and effect relationship of VD levels with certain diseases has been inconsistent [3,4,5,6,7,8,15]. In some instances, VD deficiency is believed to be a consequence of ill health rather than its cause. Nevertheless, numerous observational studies have suggested a potential role of VD in the development of various infectious diseases [16,17,18,19,20]. A plausible mechanism for the causal relationship between VD status and the progression of certain infectious diseases involves either direct or indirect interactions of the components of the VD pathway with the host immune system [18,19,20]. In support of this view, VDRs’ expression was confirmed in several white blood cells, such as monocytes and activated T and B lymphocytes [20]. Besides, the production of 1,25(OH)_2_D_3_ by local immune cells in response to infection has also been reported [21]. This paracrine and intracrine effect of VD on the immune system appears to be distinct from its classical endocrine effects on calcium regulation [18]. In line with this, the VD-induced production of the antimicrobial peptide, cathelicidin, plays a critical role in the innate defense system against several bacterial and viral pathogens [17,20]. Furthermore, the anti-proliferative effects of VD and its ability to induce autophagy and apoptosis were implicated in the active control of certain infections [18,19]. Likewise, the immunoregulatory functions of VD included the suppression of innate immune responses through the downregulation of inflammatory cytokines and the polarization of the adaptive immune system toward Th-2 responses [18]. However, these immune-related properties of VD seem to be very dependent on the nature of interactions between hosts and pathogens. Therefore, the true roles of VD and VDRG in the development and control of infectious diseases are still elusive [16,17].

To gain a better understanding and resolve the current controversy regarding the relationship between VD and the development and control of viral infections, I conducted an extensive literature search by using relevant keywords on the PubMed website. Based on the volume of hit papers related to a certain viral infection, I summarized and compared the effects of VD and VDRG polymorphism on the infection, pathogenesis, and treatment outcomes of key viral diseases (Figure 1). They are caused by the viruses of clinical importance, such as hepatitis C virus (HCV), respiratory viruses (influenza virus, respiratory syncytial virus (RSV), and rhinovirus), hepatitis B virus (HBV), human immunodeficiency virus (HIV), herpesviruses (herpes simplex virus type 1 (HSV 1), Epstein Barr virus (EBV), varicella-zoster virus (VZV), and the human cytomegalovirus (HCMV)), dengue virus (DENV), rotavirus, and human papillomavirus (HPV). Based on this information, the potential reasons for data inconsistency regarding the effects of VD on viral infections were analyzed. The nutritional and therapeutic values of VD in the management of these viral diseases were also reassessed. Finally, I proposed the future research direction to resolve the controversy on the role of VD and VDRG in the pathogenesis of viral diseases.

## 3. HCV

### 3.1. Effect of VD on HCV Infection

Hepatitis C is an inflammatory liver disease characterized by infection of hepatocytes with a hepatotropic, single-stranded RNA virus known as HCV. Approximately 170 million people are estimated to be infected with HCV worldwide [22]. The development of many effective, direct-acting antiviral agents that target the viral replication complex renders most HCV infections from different genotypes curable via pharmacological intervention.

Three research groups demonstrated the inhibition of HCV production by treatment of 1,25(OH)_2_D_3_ by using Huh 7.5 cells infected with HJ3-5 and JFH-1 HCV (Table 1) [23,24,25]. Matsumura et al. also observed a reduction in HCV core antigen production with 25(OH)D_3_ treatment at the extra- and intracellular levels in an in vitro system [26]. Gutierrez et al. and Lin et al. reported the anti-HCV replication activities of VD derivatives including VD_2_, VD_3_, and 1,25(OH)_2_D_3_ at 1–5 µM concentrations in vitro [27,28]. Structurally related VD analogs, such as calcipotriol and tacalcitol, also suppressed HCV replication in vitro [29]. Mechanistically, the activation of the interferon (IFN) signaling pathway, the blockade of peroxisome proliferator-activated receptor (PPAR), and the inhibition of endoplasmic reticulum-associated degradation (ERAD) pathways by VD were proposed as their modes of action [23,28]. Besides, a causal linkage between reduced apolipoprotein expression caused by 25(OH)D_3_ treatment and reduced HCV particle production was documented [24]. Ravid et al. also provided mechanistic evidence for the VDR-independent antiviral action by 25(OH)D_3_ [25]. Upon cotreatment with IFN, an enhanced inhibitory effect of 1,25(OH)_2_D_3_ on HCV replication was observed [30]. Mechanistically, this improvement in the antiviral activity of IFNα by 1,25(OH)_2_D_3_ seems to be mediated by the non-genomic action of VD since 1,25(OH)_2_D_3_ was shown to increase the binding of Stat1 to its DNA target and the subsequent upregulation of IFNα target gene expression [30]. This could be a plausible explanation for the association of VD-deficiency to poor responsiveness to PEG-IFNα treatment [31]. In clinical settings, VD deficiency (plasma 25(OH)D_3_ <20 ng/mL) was one of the most common symptoms among HCV patients, and a negative correlation between VD levels and viral loads in HCV patients was also frequently reported [32,33]. Thus, VD deficiency has been regarded as a risk factor for HCV infection and subsequent chronic progression [34]. In line with this observation, an inverse correlation between VD plasma levels and the expression of the VDR was also reported in HCV patients [35]. In contrast, one clinical study found no significant association of VD deficiency with poor virological characteristics in HCV patients [36].

### 3.2. Effect of VD on HCV Pathogenesis

Chronic liver inflammation and the subsequent progression to liver cirrhosis and hepatocellular carcinoma (HCC) are typical clinical symptoms of chronic HCV infection. A significant inverse relationship between the severity of inflammatory fibrosis and cirrhosis and serum 25(OH)D_3_ levels was observed in HCV patients [32,33,37,40,62,63,64]. This implies VD deficiency as an independent predictor and risk factor for inflammatory liver complications in HCV patients [38,39,65,66,67]. VD deficiency was also associated with reduced hepcidin expression, mixed cryoglobulinemia, and hepatic encephalopathy in HCV patients [41,42,43]. Therefore, VD supplementation has been suggested as a potential therapeutic option to reverse the hepatic complications caused by HCV infection [53]. Indeed, VD supplementation was able to suppress hepatic fibrosis induced by HCV infection [53]. One of the mechanisms for VD-dependent alleviation of hepatic fibrosis was via the VDR-dependent downregulation of the transforming growth factor (TGF) *β*-1/small mothers against decapentaplegic (SMAD) 3 pathway [68]. One clinical study showed a significantly higher mortality rate in HCV patients with severe VD deficiency (25(OH)D_3_ levels <5 ng/mL) [43]. Despite these positive effects by VD on HCV pathogenesis, several research groups also reported seemingly contradictory results. For example, treatment-naive Chinese HCV patients failed to show any correlation between serum levels of 25(OH)D_3_ and 24,25(OH)_2_D_3_ and liver fibrosis [54]. Oliveira et al. and Esmat et al. also reported no significant association between VD serum levels and inflammatory activity or the degree of liver fibrosis [55,59]. Another clinical study also suggested that VD deficiency was not responsible for poor virological characteristics in HCV patients [36]. In support of this view, VD supplementation had no immediate impact on the viral RNA loads of HCV patients [61].

### 3.3. Effect of VD on HCV Infection Treatment Outcomes

Before the development of direct-acting antiviral therapy that targets viral structural proteins, a pegylated (PEG)-IFNα/ribavirin combination therapy was the standard of care for HCV patients. Several researchers reported a positive correlation between VD status and sustained virological response (SVR) rates to this standard PEG-IFNα/ribavirin therapy for HCV patients [34,48,51,52,66]. Therefore, VD deficiency was regarded as a potential predictor of an unfavorable response to IFNα-based HCV treatment [45,46,50,64]. In line with this, VD supplementation improved the probability of achieving an SVR following antiviral treatment [37,45,46,49,51,69,70,71]. HIV–HCV coinfected patients also demonstrated a strong correlation between VD levels and SVR [47]. These data strongly emphasized the utility of VD supplementation for the management of HCV infection in conjunction with standard IFNα-based therapy [35]. However, contrary to these results, many reports failed to observe a significant association between VD levels and SVR to IFNα-based therapy in chronic HCV infections [56,57,58,59,60,72]. In particular, a specific ethnic group such as African American HCV patients also did not show any relationship between 25(OH)D_3_ serum concentration and SVR [73]. Furthermore, two other studies also failed to establish a relationship between 25(OH)D_3_ levels, biochemical liver markers, and fibrosis stages in HCV patients [56,61].

### 3.4. Effect of VDRG Polymorphisms on HCV Infection

Three studies examined the potential effects of VDRG polymorphisms on HCV infection. Wu et al. demonstrated the protective roles of VDR variants including rs7975232-C, rs2239185-T, rs11574129-T, rs757343-A, rs739837 A, and CYP24A1 variant rs6068816-T against HCV infection [34]. On the other hand, VDBP variants (rs7041-G and rs3733359-T), a CYP24A1 variant (rs6013897-A), and CYP2R1 variants (rs12794714-G, rs10741657-A, rs1562902-C, and rs10766197-G) were associated with increased susceptibility to HCV infection [13]. Besides, HCV patients with VDR variants such as bAt [CCA]-haplotype, *ApaI* CC genotype, and *TaqI* AA genotype had higher viral loads [74].

### 3.5. Effect of VDRG Polymorphisms on HCV Pathogenesis

Since VDRG plays a key role in the maintenance of proper plasma VD concentration, many studies aimed to identify the potential effects of VDRG polymorphisms on viral diseases. Seven studies have reported potential VDRG polymorphism effects on HCV pathogenesis. Among HCV patients, VDBP protein levels were significantly higher for normal/mild fibrosis when compared to those for advanced fibrosis [75]. VDR *BsmI* and *TaqI* variants affected the progression of fibrosis in HCV patients [76]. When Barooach et al. determined whether VDR, VDBP, and CYP2R1 gene polymorphisms are risk factors for clinical complications in HCV-related HCC patients, they identified the VDR *ApaI* CC genotype and the VDR bAt haplotype as independent predictors for cirrhosis and HCC development in HCV patients [77]. When Langer et al. performed a similar study to investigate the potential link between CYP2R1, VDBP, and DHCR7 genotypes and the risk of HCV-related HCC development by using 1279 HCV-related HCC patients, they found a functionally relevant role for VD in the prevention of HCV-related HCC [44]. This further implies that defective signaling in the VD pathway may contribute to hepatocarcinogenesis in HCV-infected patients [44]. Although the detailed mechanistic explanation for this linkage of VDR, VDBP, and CYP2R1 polymorphisms to the development of hepatocellular carcinoma was not given, the hepatic conversion of VD to 23(OH)D3, the plasma transportation of VD by VDBP, and the intracellular binding of VD with VDR might be affected by polymorphic changes in VDR, VDBP, and CYP2R1 genes. These changes might, in turn, alter the overall strength of VD signaling, ultimately affecting the development of HCC in HCV patients. VDR *FokI* rs2228570 TT/TC genotypes were also suggested as risk factors for advanced liver fibrosis in HCV patients [78]. In addition, the expression levels of VDR in cholangiocytes were inversely correlated with pathological progression markers of HCV infection [79]. Moreover, CYP2R1 expression in hepatocytes showed a strong correlation with VDR levels. In line with this, VDR expression levels demonstrated a negative correlation with the severity of liver histology in both non-alcoholic steatohepatitis and HCV patients [79]. Interestingly, rs7041 and rs4588 VDBP polymorphisms did not play a direct role in liver fibrosis despite their strong association with VD levels in HCV patients [80].

### 3.6. Effect of VDRG Polymorphisms on HCV Infection Treatment Outcomes

As critical determinants of VD levels, the polymorphic effects of VDRG on the responsiveness of HCV treatment in HCV patients were also studied. Six studies examined the potential effects of VDRG polymorphisms on HCV infection treatment outcomes. Cusato et al. showed that CYP27B1 rs10741657, CYP24A1, VDR rs2228570, *FokI*, and *TaqI* polymorphisms, in combination with the IL28B polymorphism (rs12979860), impacted HCV infection treatment outcomes [81]. DHCR7-TT and rs12785878 polymorphisms were also significantly associated with an SVR to IFN-based therapy [57]. A polymorphism near the CYP27B1-1260 promoter region (rs10877012) reduced 1,25(OH)_2_D_3_ serum levels, which, in turn, worsened an SVR in HCV patients [82]. Falleti et al. showed that a combination of a basal VD level >20 ng/mL and VDBP wild type 1 was an independent predictor of a higher SVR to IFN-based therapy [83]. Petta et al. also demonstrated an association between 25(OH)D_3_ serum levels, IL28B status, and the probability of achieving an SVR [84]. On the other hand, the VDR bAt (CCA) haplotype, consisting of the *BsmI* rs1544410 C, *ApaI* rs7975232 C, and *TaqI* rs731236 A alleles, was linked with a poor response to IFNα-based therapy [78]. However, there are conflicting results regarding the roles of VDRG polymorphisms on HCV infection treatment outcomes. For example, VDR gene polymorphism did not correlate with rapid virological response and SVR achievement in HCV patients [74]. In addition, the VDBP, CYP2R1, and CYP27B1 polymorphisms did not play any role in the treatment outcomes of HCV infection [85].

## 4. Respiratory Viruses Such as Influenza Virus, RSV, and Rhinovirus

### 4.1. Effect of Vd on Respiratory Viral Infections, Immune Response, and Pathogenesis

Respiratory viruses are defined as viruses responsible for the development of either upper or lower respiratory tract infections. Typically, they include influenza virus, parainfluenza virus, adenovirus, respiratory syncytial virus (RSV), and rhinovirus. With regards to the role of VD in control of viral respiratory infections, VD inhibited rhinovirus replication in primary cystic fibrosis bronchial cells through the induction of cathelicidin [86] (Table 2). Besides, VD also decreased rhinovirus replication and release and increased the expression of IFN-stimulated genes and cathelicidin in human primary brain endothelial cells (HPBEC) infected with rhinovirus 1B [87]. In particular, cathelicidin exhibited direct anti-rhinovirus activity in in vitro experiments using HPBEC cells infected with rhinovirus 1B [87]. In line with this, the pretreatment of A549 respiratory epithelial cells with 25(OH)D_3_ induced transient resistance to rhinovirus infection [88]. It also attenuated the rhinovirus-induced expression of genes encoding intercellular adhesion molecule 1 and platelet-activating factor receptor in A549 cells infected with rhinovirus 16 [88]. The induction of IkBa, an NF-kB inhibitor, via VD treatment attenuated the expression of NF-kB-driven proinflammatory genes in human tracheobronchial epithelial cells (hTBE) infected with RSV strain 2A [89]. This, in turn, decreased the inflammatory response to rhinovirus infection in airway epithelium without affecting viral clearance [89]. Another research group demonstrated VD treatment-induced inhibition of NFκB and STAT1-regulated gene expression in RSV-infected A549 cells [90]. However, despite the positive effects displayed by VD on respiratory viruses, in vitro VD treatment had no direct effect on rhinovirus replication in experiments using primary human bronchial epithelial cells (hBEC) [91]. Moreover, the VD-induced production of cathelicidin had no impact on the burden of influenza virus infection [92]. Fitch et al. also failed to observe any VD-induced anti-inflammatory effects during RSV infection in experiments using fresh peripheral blood mononuclear cells (PBMC) or CD4+ monocytes [93].

VD deficiency has been associated with an increased risk of lower respiratory viral infections caused by the influenza virus, RSV, and rhinovirus [100]. In particular, preschool females with low 25(OH)D_3_ levels were more susceptible to respiratory viral infections than males [94]. Moreover, low VD levels were associated with a significantly elevated risk of intensive care unit admission and invasive mechanical ventilation after respiratory viral infections [95]. In line with this, a higher 25(OH)D_3_ cord serum level reduced the risk of virally induced wheezing in a clinical study of 190 affected children [96]. As expected, VD supplementation protected against respiratory viral infections in both healthy patients and patients with respiratory diseases through the positive modulation of innate immune responses [101]. The meta-analysis of several clinical studies by Martineau et al. also showed the reduced risk of acute respiratory tract infection by VD supplementation [102]. In support of this, higher VD levels also correlated with increased immunogenicity, which was demonstrated by the more efficient production of influenza virus-specific antibodies [97]. However, despite numerous evidence supporting the positive role of VD in the prevention of respiratory viral infections, several studies reported seemingly conflicting results. For example, no significant association between VD levels and immunogenic responses to influenza vaccination was found by one clinical study [103]. Besides, no evidence of improved influenza vaccine immunogenicity with VD supplementation was reported in HIV-positive populations [98]. VD supplementation also failed to induce consistent protective effects on respiratory viral infections caused by the influenza virus, RSV, or rhinovirus [104]. VD supplementation was even found to be associated with an increase in repeat episodes of infectious pneumonia [105]. Finally, VD deficiency did not play any role in the development of acute bronchiolitis caused by RSV infection in 145 infants [99].

### 4.2. Effect of VDRG Polymorphisms on Respiratory Viral Infections

A significant association between VDR *FokI* polymorphism and respiratory viral infections has been observed [106]. In line with this, VDR *FokI* variants also showed enhanced immunopathology and exacerbated bronchiolitis after RSV infection [90]. This effect was mediated by blocking STAT1-mediated antiviral immune reactions to RSV infection [90]. Three single nucleotide polymorphisms (SNP) in VDR (rs4334089, rs11568820, and rs7970314) and one SNP in CYP3A4 (rs2740574) were also suggested as risk factors for upper respiratory infection by rhinovirus and RSV [107]. This is supported by McNally et al. who reported an association between the VDR *FokI* polymorphism and the severity of RSV infection [108]. The VDBP’s haplotype also played a positive role in RSV bronchiolitis in infancy and subsequent asthma development [109]. Besides, the VDBP’s haplotype was associated with higher VDBP levels, which might have detrimental effects on RSV bronchiolitis due to reduced levels of VD [109].

## 5. HBV

### 5.1. Effect of VD on HBV Infection, Immune Response, Pathogenesis, and Treatment Outcomes

More than 240 million people around the world live with chronic HBV infection [110,111]. Reverse transcriptase inhibitors along with nucleoside and nucleotide structures are the main treatments against HBV infection [112]. However, similar to HIV patients, these anti-HBV therapeutics are not able to provide a complete cure for HBV infection because of their inability to remove stable, nuclear, and covalently closed circular DNA (cccDNA), which serves as a transcription template for the continuous production of viral mRNA and pre-genomic RNA [113].

According to epidemiological studies, significantly decreased VD levels were consistently found in chronic HBV patients [114]. The 25(OH)D_3_ plasma level showed a significant inverse correlation with plasma HBV DNA loads and a positive correlation with the seroclearance of the hepatitis B surface antigen (HBsAg) [115,116] (Table 3). In turn, reduced VD levels were associated with the clinical progression of liver cirrhosis and adverse clinical outcomes [117]. In line with this clinical evidence, the downregulation of VDR expression in HBV-transfected cells caused an increase in HBV transcription and translation [118]. Effective antiviral therapy, in contrast, restored VD to normal levels in HBV patients [119]. Among 560 HBV patients, those with sufficient VD achieved a higher SVR than those with VD deficiency [120]. Besides, the addition of VD to standard IFNα therapy achieved higher efficacy than IFNα alone in an HBV transgenic mouse model [121]. However, several studies also reported contradictory results regarding the effects of VD on HBV infection. Berkan-Kawinska et al. reported no association between 25(OH)D_3_ deficiency and poor virological characteristics in 35 HBV patients [36]. In support of this, HBV DNA levels were not associated with VD levels in 84 HBV patients [122]. In addition, serum 25(OH)D_3_ concentration did not correlate with histological or biochemical markers of liver inflammation in 58 HBV patients [123]. Serum 25(OH)D_3_ also failed to have any impact on the immune control of HBV infection [123]. 25(OH)D_3_ levels did not affect the immunogenicity of hepatitis B e antigen (HBeAg), HBV viral loads, or fibrosis stage in two studies examining 560 and 242 HBV patients, respectively [120,124]. Finally, baseline VD levels were not associated with HBV infection treatment outcomes with a tenofovir plus PEG-IFNα combination therapy in 737 HBV patients [125].

### 5.2. Effect of Vdrg Polymorphisms on Hbv Infection, Pathogenesis, and Treatment Outcomes

Eight studies examined the potential effects of VDRG polymorphisms on HBV infection, pathogenesis, and treatment outcomes. A VDR gene polymorphism at codon 352 (genotype tt) was significantly underrepresented among HBV patients [127]. In particular, the VDR *FokI* FF gene was shown to be a risk factor for HBV infection [128]. In addition, VDR gene polymorphisms were associated with distinct clinical phenotypes in Taiwanese HBV patients [129]. In line with this, VDR *ApaI* was also associated with the clinical outcome of and liver disease progression in HBV patients [130]. From a therapeutic perspective, VDR rs7975232/*ApaI* was identified as a pretreatment predictor of sustained HBsAg seroclearance in HBeAg-positive HBV patients when treated with PEG-IFNα [131]. In support of this, VDR *ApaI* SNP was associated with viral loads and the presence of HBsAg in response to PEG-IFNα treatment [132]. VDR *FokI* SNP and the bAt haplotype were also suggested as independent factors that can predict PEG-IFNα treatment responses in HBV patients [133]. Finally, the VDBP rs222020 TT genotype independently predicted the sustained HBsAg seroclearance and the normalization of aspartate aminotransferase after PEG-IFNα treatment [134].

## 6. HIV

### 6.1. Effect of VD on HI Infection, Immune Response, Pathogenesis, and Treatment Outcomes

HIV is an etiological agent responsible for the development of acquired immunodeficiency syndrome (AIDS). Concerning the effects of VD on HIV biology, 1,25(OH)_2_D_3_ inhibited HIV replication through the induction of autophagy and phagosomal maturation in PBMC infected with HIVBa-L [135,136] (Table 4). Mechanistically, the induction of cathelicidin by VD played an essential role in 1,25D_3_-induced autophagic flux and inhibition of HIV replication [135]. In particular, high levels of VD and its receptor are related to natural resistance to HIV-1 infection in the PBMC from HIV-1-exposed seronegative individuals [137]. In line with this, VD deficiency was suggested as a predictor for short-term mortality in 250 HIV patients [138]. The importance of cathelicidin in the suppression of HIV infection by VD was further supported by elevated cathelicidin mRNA levels in PBMCs and oral mucosa of HIV-1-exposed seronegative individuals [139]. Among 90 patients with HIV/Kaposi’s sarcoma, HIV RNA levels were significantly higher among those with VD deficiency [140]. VD deficiency was also associated with reduced recovery of CD4+ T-cell count in 398 HIV patients by anti-retroviral therapy [141]. In support of this, VD supplementation attenuated HIV-1 replication and increased the number of circulating leukocytes from 100 VD_3_-fed healthy adults [142]. In in vitro experiments using PBMC infected with HIVBa-L, VD treatment reduced HIV-1 infection in T cells by inducing antiviral gene expression and reducing the levels of viral co-receptor CCR5 [143]. In ex vivo experiments using PBMC from seronegative individuals infected with HIV-1, VD treatment was able to reduce HIV-1 transmission by specifically modulating the activation of T cells and the production of antiviral factors [144]. VD supplementation also reduced TB/HIV co-infection and its progression [145]. Cathelicidin levels were significantly lower in HIV patients when compared to healthy controls [146]. Besides, decreased 25(OH)D_3_ levels were also linked to reduced responsiveness to retroviral therapy [147]. However, despite all these positive effects of VD on HIV infection, several research groups reported opposing results. VD_3_ and 1,25(OH)_2_D_3_ administered over 16 weeks failed to change T cell numbers in HIV patients [148]. In addition, no HIV-dependent variables were found to be associated with 25(OH)D_3_ levels in HIV patients [149]. Besides, no improvements in HIV viral loads, CD4+ T cell counts, or CD8+ T cell counts were detected after VD supplementation in HIV patients [150]. Moreover, routine multiple micronutrient supplementation, including VD, showed no significant beneficial effects on mortality in HIV patients [151]. In the therapeutical perspective, VD deficiency was not associated with the outcomes of antiretroviral therapy in HIV patients [140].

### 6.2. Effect of VDRG Polymorphisms on HIV Infection and Pathogenesis

Three studies reported results regarding the potential effects of VDRG polymorphisms on HIV infection, pathogenesis, and treatment outcome. VDR diplotypes in 5′ untranslated region (UTR) and 3′UTR locus combinations were associated with the pathogenesis of AIDS [154]. The VDR variant rs1544410_AA was also associated with the progression to AIDS and resistance to HIV-1 [155]. Moreover, VDR haplotypes influenced the risk of HIV-1 acquisition [156].

## 7. Herpesviruses

More than 90% of the adult population is infected with one or more forms of herpes virus [157]. Herpesviruses, including herpes simplex virus type (HSV) 1, Epstein Barr virus (EBV), varicella-zoster virus (VZV), and human cytomegalovirus (HCMV), are responsible for a wide variety of recurrent diseases, such as cold sores, shingles, congenital defects, and several malignancies. Concerning the effects of VD on herpesvirus infections, a significant downregulation of HSV-1 titers was observed in both 25(OH)D_3_- and 1,25(OH)_2_D_3_-treated HeLa cells infected with HSV-1 [158] (Table 5). VDR expression was downregulated in human foreskin fibroblasts infected with CMV AD169 [159]. In line with this, among 547 HCMV patients, VD deficiency was independently associated with an increased incidence of opportunistic viral infection by HCMV [160]. A total of 88 chronic hemodialysis patients with VD deficiency had significantly reduced immunogenicity against VZV infection [161]. A significant correlation between VD deficiency and the incidence of HCMV disease was also reported by a clinical study examining the 139 HCMV patients [162]. In addition, VD deficiency was also suggested as a risk factor for acute rejection caused by HCMV infection after kidney transplantation [163]. VD deficiency was also frequently observed in infectious mononucleosis patients caused by EBV infection [164]. Along with an inverse correlation between 25(OH)D_3_ levels and EBV infection [165], a significant association between low VD serum levels and the presence of recurrent herpes labialis was also reported [166]. However, in spite of these positive results, VD metabolites failed to inhibit HCMV replication in human foreskin fibroblasts infected with CMV AD169 [159].

## 8. Dengue Virus

Dengue virus (DENV) is responsible for the development of dengue fever, a mosquito-borne tropical disease. A high dose of 1,25(OH)2D_3_ had an immunoregulatory role in reducing inflammation during DENV infections in U937-DC-SIGN and THP1 macrophages infected with DENV-2 [167]. Mechanistically, the suppression of inflammatory cytokine response by VD treatment during DENV infection was mediated via the toll-like receptor (TLR) 4/NF-κB/miR-155-5p/suppressor of cytokine signaling (SOCS)-1 axis [179]. Mouse monocyte-derived macrophages differentiated in the presence of VD restrict DENV infection and moderate the classical inflammatory cytokine response caused by DENV infection [168] (Table 5). This VD-driven differentiation led to the reduced surface expression of C-type lectins, including the mannose receptor that acts as a primary receptor for the DENV attachment on macrophages [168]. Besides, macrophages, which were derived from VD-supplemented healthy donors, exhibited higher resistance to DENV infection, significantly decreased levels of pro-inflammatory cytokines, and increased levels of immunosuppressive cytokines like IL-10 [169]. High-dose VD administration also decreased DENV infection in monocyte-derived dendritic cells from 30 VD_3_-fed individuals [170]. However, paradoxically, low serum 25(OH)D_3_ concentrations in dengue fever patients reduced the pathological progress into dengue hemorrhagic fever/dengue shock syndrome [171]. In line with this, circulating VD is much higher during an acute dengue episode than during disease-free periods. [171]. Regarding the effects of VDRG polymorphisms on DENV infection, 3′ UTR haplotypes of the VDR gene were differentially associated with the risk of clinical symptoms caused by DENV infection [180].

## 9. Rotavirus

Rotaviruses are one of the most common causes of virus-induced diarrheal diseases among infants and young children. With respect to the effects of VD on rotavirus, physiological concentrations of 25(OH)D_3_ decreased porcine rotavirus replication in intestinal porcine enterocytes (IPEC-J2) cells [172] (Table 5). In addition, a study of 70 rotavirus patients suggested an association of VD deficiency with rotaviral diarrhea [173]. VD_3_ was also able to attenuate rotavirus infection by regulating autophagic maturation and cathelicidin gene expression in IPEC-J2 cells infected with porcine rotavirus [174]. 1,25(OH)_2_D_3_ also alleviated rotavirus infection through the miRNA-155-5p-mediated regulation of the TANK-binding kinase (TBK)1/interferon-responsive factor (IRF) 3 signaling pathway [175]. In line with this, VD supplementation alleviated intestinal damage and protected against inflammation in pigs infected with porcine epidemic diarrhea virus (PEDV) [176].

## 10. Human Papillomavirus (HPV)

High-risk type HPV infection is responsible for the development of cervical cancers in women. According to a study examining 2353 sexually active women, HPV prevalence was associated with VD deficiency [177] (Table 5). However, in contrast to this, Garcia-Carrasco et al. found no association between VD deficiency and the development of cervical cancers in 67 HPV-positive women with systemic lupus erythematosus [178].

## 11. Reasons for the Controversies and Future Research Direction

For the successful establishment of viral infection and subsequent pathogenesis in the host, a virus needs to overcome numerous hurdles that are imposed by a host immune system. Therefore, the evolution of the immune evasion strategy by a virus is an absolute requirement for its maximal survival inside the host. As shown previously, the biological functions of VD seem to be in intricate association with the many aspects of the host physiology. In particular, the non-skeletal, immune-related functions of VD begin to be explored in the context of different pathological conditions induced by viral infections [18,19,20]. The discovery of VDRs’ expression in several white blood cells [20] and the production of 1,25(OH)_2_D_3_ by local immune cells in response to infection further support the potential role of VD in the control of pathological viral infections [21]. In particular, the VD-induced production of cathelicidin is one of the best characterized immune-related functions of VD for the control of bacterial and viral infections [17,20]. Furthermore, the direct regulation of autophagy and the apoptosis of immune cells by VD seems to be one of the immune-regulatory actions of VD required for the clearance of the viral infections [18,19]. However, in spite of all this progress, our knowledge in this filed is still lagging far behind compared to the already known immunological functions of other essential nutrients, further complicating the deciphering of the cause and effect relationship of the VD biology in the development of many viral diseases [3,4,5,6,7,8,15]. On top of this, many different variables seem to serve as contributing factors to this added inconsistency and discrepancy surrounding the biological roles of VD in the context of viral infections. They include in vitro and in vivo variability, differences in experimental systems, differences in the criteria used for defining patients with a certain viral infection, and differences in clinical trial design. This might be one of the ultimate reasons for all these controversial effects of VD on the infection, pathogenesis, and treatment outcomes of several viral diseases. Therefore, we need a more complete picture of the diverse biological activities of VD in the context of different viral infections. For this goal, more systematic and thorough in vitro as well as in vivo preclinical studies should be performed before attempting the clinical application of VD. More validated markers for the VD-dependent immunological modulation will be necessary for a more accurate application of the beneficial effects of VD to virally infected patients. In particular, due to the seasonal and dietary fluctuation of the plasma VD concentrations among patients, more careful attention needs to be paid to determine the optimal VD concentrations required for the maximal beneficial effects on either the prevention or control of viral infections. For a successful translation of in vitro experimental data into in vivo, the only physiologically achievable concentrations of VD should be tested in an in vitro system. Due to the lack of coexisting immune systems in most in vitro cell experiments, more relevant immune-related functions of VD need to be explored by using a variety of animal infection models with an intact immune system. Instead of the random empirical approaches, which formed the basis for most VD clinical trials so far, the nutritional and clinical application of VD in the management of viral infections should be based on a more solid scientific rationale that will be only formed by more hypothesis-driven research in the future.

## 12. Conclusions

In this review, an extensive summary was generated regarding the diverse effects of VD and VDRG on the infection, pathogenesis, and treatment outcomes of several diseases caused by many clinically relevant viruses. Despite many exciting and promising results, an extensive literature review demonstrates that there is no consensus on the roles of VD and VDRG in the development and control of various diseases caused by viral infections. The full picture of the immunomodulatory functions of VD and VDRG in the context of different viral infections is still being formed. Therefore, more thorough and systematic in vitro, in vivo, and clinical studies are necessary to fully harness the potential preventive and therapeutic effects of VD and VDRG at controlling infectious diseases. Until then, it would be premature to provide recommendations on the use of VD for virally infected patients.

## Figures and Tables

**Table 1:** Effects of VD on hepatitis C virus (HCV) infection, pathogenesis, and treatment outcomes.

Reference	Experimental System	VD Tested or Status	VD Conc	Effects	Description of Effects
[23]	HJ3-5 HCV in Huh 7.5 cells	VD_3_	2.5 µM	Positive	Inhibition of HCV production
[24]	JFH-1 HCV in Huh 7.5 cells	VD derivatives	1–10 µM	Positive	
[25]	HJ3-5 HCV in Huh 7.5 cells	VD_3_ and 25(OH)D_3_	5 µM	Positive	
[26]	JFH-1 in Huh 7.5 cells	25(OH)D_3_	0.95 µM	Positive	Reduction of HCV core antigen production
[27]	BM4-5 FEO and SGR-JFH1/Luc in Huh 7.5 cells	1,25(OH)_2_D_3_	1–5 µM	Positive	Inhibition of HCV replication
[28]	NS3 protease-based SEAP reporter in Huh 7.5 cells	1,25(OH)_2_D_3_	1 µM	Positive	
[29]	Subgenomic GT 1b replicon and JFH1 in Huh 7.5 cells	Calcipotriol and tacalcitol	10 nM	Positive	
[32]	50 GT 4 patients	25(OH)D_3_ deficiency	15 ng/ml	Positive	Increased HCV viral RNA loads Increased liver inflammation, fibrosis, and cirrhosis
[33]	50 GT 4 patients	25(OH)D_3_ deficiency	<20 ng/ml	Positive	
[37]	191 GT 1 patients	25(OH)D_3_ deficiency	18 ng/ml	Positive	
[38]	331 mixed GT patients	25(OH)D_3_ deficiency	27.5 ng/ml	Positive	
[39]	86 HIV/HCV-coinfected patients	25(OH)D_3_ deficiency	18.2 ng/ml	Positive	
[40]	58 patients	25(OH)D_3_ deficiency	<30 ng/ml	Positive	
[41]	50 child patents	25(OH)D_3_ deficiency	13.72 ng/ml	Positive	Reduced hepcidin expression
[42]	106 GT 1 and 2 patients	25(OH)D_3_ deficiency	5.61 ng/ml	Positive	Mixed cryoglobulinemia
[43]	135 cirrhosis patients	25(OH)D_3_ deficiency	6.81 ng/mL	Positive	Hepatic encephalopathy and higher mortality rate
[44]	496 HCC patients	25(OH)D_3_ deficiency	12.7 ng/ml	Positive	Increased hepatocellular carcinoma
[45]	211 patients	25(OH)D_3_ deficiency	<10 ng/ml	Positive	Decreased SVR to PEG-IFNα/ribavirin therapy
[46]	50 GT 2 and 3 patients	25(OH)D_3_ deficiency	<15 ng/ml	Positive	
[47]	65 HIV coinfected patients	25(OH)D_3_ deficiency	<10 ng/ml	Positive	
[48]	177 GT 1b patients	25(OH)D_3_ deficiency	<18 ng/ml	Positive	
[34]	898 patients	25(OH)D_3_ deficiency	<20 ng/ml	Positive	
[49]	72 GT1 patients	25(OH)D_3_ supplementation	37 ng/ml	Positive	Increased SVR to PEG-IFNα/ribavirin therapy
[45]	42 patients	25(OH)D_3_ supplementation	>20 ng/ml	Positive	
[46]	50 GT 2 and 3 patients	25(OH)D_3_ supplementation	34 ng/ml	Positive	
[50]	84 GT 1b patients	25(OH)D_3_ supplementation	19.6 ng/ml	Positive	
[51]	66 child patents	25(OH)D_3_ supplementation	65.26 nmol/L	Positive	
[52]	36 GT1 patients	25(OH)D_3_ supplementation	39.6 ng/ml	Positive	
[53]	58 patients	25(OH)D_3_ supplementation	45.6 ng/ml	Positive	Decreased liver fibrosis
[54]	122 patients	25(OH)D_3_ and 24,25(OH)_2_D_3_ deficiency	5.84 ng/mL and 1.78 ng/ml	Negative	No effect on fibrosis
[55]	74 patients	25(OH)D_3_ deficiency	<20 ng/ml	Negative	No effect on liver inflammation and fibrosis
[36]	90 patients	25(OH)D_3_ deficiency	18.8 ng/ml	Negative	No effect on virological characteristics
[56]	274 GT 1 patients	25(OH)D_3_ deficiency	76.6 nmol/L	Negative	No effect on SVR to PEG-IFNα/ribavirin therapy
[57]	398 GT 1 patients	25(OH)D_3_ deficiency	18.7 ng/ml	Negative	
[58]	1145 non-responders	25(OH)D_3_ deficiency	<20 ng/ml	Negative	
[59]	101 GT4 patients	25(OH)D_3_ supplementation	65.86 ng/ml	Negative	No effect on SVR to PEG-IFNα/ribavirin therapy
[60]	32 non-responders	25(OH)D_3_ supplementation	66 ng/ml	Negative	
[56]	274 GT 1 patients	25(OH)D_3_ deficiency	76.6 nmol/L	Negative	No effect on liver inflammation and fibrosis
[61]	108 patients	25(OH)D_3_ deficiency	<20 ng/ml	Negative	
[61]	37 patients	25(OH)D_3_ supplementation	30–93 ng/ml	Negative	No effect on HCV viral RNA loads

Abbreviations used are as follows. GT; genotype, JFH; Japanese fulminant hepatitis, SGR; subgenomic replicon, SEAP; secretory alkaline phosphatase, SVR; sustained response rate, HCC; hepatocellular carcinoma, PEG; polyethylene glycol.

**Table 2:** Effects of VD on respiratory viral infection, pathogenesis, and treatment outcomes.

Reference	Experimental System	VD Tested or Status	VD Conc	Effects	Description of Effects
[86]	Primary cystic fibrosis cells infected with rhinovirus 16	1,25(OH)_2_D_3_	0.1 µM	Positive	Inhibition of rhinovirus replication
[87]	HPBEC cells infected with rhinovirus 1B	1,25(OH)_2_D_3_	>0.1 µM	Positive	Decreased rhinovirus replication and release
[88]	A549 cells infected with rhinovirus 16	25(OH)D and 1,25(OH)_2_D_3_	0.1 µM	Positive	Resistant to rhinovirus infection
[89]	hTBE cells infected with RSV strain 2A	1,25(OH)_2_D_3_	0.1 µM	Positive	Suppression of RSV-induced inflammation
[94]	95 preschool children	25(OH)D deficiency	50 nmol/L	Positive	More vulnerable to respiratory viral infections
[95]	90 hospitalized children	25(OH)D deficiency	32 ng/ml	Positive	More intensive care unit admission and invasive mechanical ventilation
[96]	190 children	Cord blood 25(OH)D deficiency	6.33 ng/ml	Positive	Increased virally induced wheezing
[97]	46 children and adults blood samples	25(OH)D deficiency	<20 ng/ml	Positive	Reduced immunogenicity to influenza vaccination
[91]	Primary hBEC cells	1,25(OH)_2_D_3_	0.01 µM	Negative	No effect on rhinovirus replication
[93]	Fresh PBMCs or CD4+ monocytes	1,25(OH)_2_D_3_	0.1 µM	Negative	No effect on RSV-induced inflammation
[98]	28 HIV-infected adults	25(OH)D supplementation	Not measured	Negative	No effect on immunogenicity to influenza vaccination
[99]	145 infants	25(OH)D deficiency	<20 ng/ml	Negative	No effect on bronchiolitis severity by RSV infection

The abbreviations used are as follows. PBMC; peripheral blood mononuclear cells, HPBEC; human primary brain endothelial cells, hTBE; human tracheobronchial epithelial cells, hBEC; human bronchial epithelial cells.

**Table 3:** Effects of VD on hepatitis B virus (HBV) infection, pathogenesis, and treatment outcomes.

Reference	Experimental System	VD Tested or Status	VD Conc	Effects	Description of Effects
[115]	173 patients	25(OH)D_3_ deficiency	22.19 ng/ml	Positive	Increased HBV DNA loads
[116]	53 patients	25(OH)D_3_ deficiency	<20 ng/ml	Positive	Decreased seroclearance or seroprotection of HBsAg
[117]	400 patients	25(OH)D_3_ deficiency	<20 ng/ml	Positive	Increased liver cirrhosis and adverse clinical outcomes
[120]	560 patients	25(OH)D_3_ deficiency	<20 ng/ml	Positive	Decreased SVR
[121]	HBV transgenic mice	25(OH)_2_D supplementation	Not measured	Positive	Increased SVR to IFNα therapy
[123]	58 patients	25(OH)D_3_ deficiency	28.81 ng/ml	Negative	No effect on liver aminotransferase, histology, or immune control of HBV
[124]	345 patients	25(OH)D_3_ deficiency	7.83 ng/ml	Negative	No effect on HBeAg or HBV viral loads
[126]	242 patients	25(OH)D_3_ deficiency	33.9 ng/ml	Negative	No effect on fibrosis stages or HBV viral loads
[36]	35 patients	25(OH)D_3_ deficiency	17.6 ng/ml	Negative	No effect on virological characteristics
[122]	84 patients	25(OH)D_3_ deficiency	32 ng/ml	Negative	No effect on HBV DNA loads
[125]	737 patients	25(OH)D_3_ deficiency	<20 ng/ml	Negative	No effect on treatment outcome

The abbreviations used are as follows. HBsAg; hepatitis B surface antigen, HBeAg; hepatitis B e antigen.

**Table 4:** Effects of VD human immunodeficiency virus (HIV) infection, pathogenesis, and treatment outcome.

Reference	Experimental System	VD Tested or Status	VD Concentration	Effects	Description of Effects
[135,136,152]	PBMC infected with HIVBa-L	1,25(OH)_2_D_3_	0.2 nM	Positive	Inhibition of HIV replication
[143]	PBMC infected with HIVBa-L	1,25(OH)_2_D_3_	0.1 µM	Positive	
[153]	PBMC from HIV-1-exposed seronegative individuals	1,25(OH)_2_D_3_	Not measured	Positive	Reduced HIV infection and resistant to HIV infection
[138]	250 patients	25(OH)D_3_ deficiency	<30 ng/ml	Positive	Increased mortality
[141]	398 patients	25(OH)D_3_ deficiency	<20 ng/ml	Positive	Lower absolute CD4+ T cell number
[142]	PBMC from 100 VD_3_-fed healthy adults infected with HIVBa-L	VD_3_ supplementation	150 nmol/L	Positive	Attenuation of HIV replication and increased leucocyte number
[140]	90 patients with HIV/Kaposi’s sarcoma	25(OH)D_3_ deficiency	<75 nmol/L	Positive	Increased HIV RNA levels
[144]	PBMC from seronegative individuals infected with HIV-1	1,25(OH)_2_D_3_	0.01 µM	Positive	Reduced HIV infection of CD4+ T cells
[147]	72 patients	25(OH)D_3_ deficiency	<20 ng/ml	Positive	Decreased responsiveness to retroviral therapy
[148]	61 patients	VD_3_ and 1,25(OH)_2_D_3_ supplementation	>70 nmol/L	Negative	No effect on T cell numbers
[150]	173 patients	VD_3_ supplementation	>75 nmol/L	Negative	No effect on HIV viral load, CD4+, cell counts, CD8+ T cell counts, body mass index or middle upper arm circumference

The abbreviations used are as follows. PBMC; peripheral blood mononuclear cells.

**Table 5:** Effects of VD on infection, pathogenesis, and treatment outcome by other viruses.

	Reference	Experimental System	VD Tested or Status	VD Concentration	Effects	Description of Effects
**Herpesviruses**	[158]	HeLa cells infected with HSV-1	25(OH)D_3_ and 1,25(OH)_2_D_3_	1 µM and 100 nM	Positive	Decreased HSV-1 titer
	[160]	547 patients	25(OH)D_3_ deficiency	<50 nmol/L	Positive	Increased incidence of opportunistic CMV infection
	[161]	88 chronic hemodialysis patients	25(OH)D_3_ deficiency	<30 ng/ml	Positive	Lower VZV-IgG
	[162]	139 patients	25(OH)D_3_ deficiency	<25 nmol/L	Positive	Increased incidence of CMV disease
	[163]	373 renal transplant recipients	1,25(OH)_2_D_3_ deficiency	<20 pg/ml	Positive	Increased acute transplant rejection caused by CMV infection
	[165]	482 with multiple sclerosis	25(OH)D_3_ deficiency	<20 ng/ml	Positive	Increased EBV loads
	[166]	50 patients	25(OH)D_3_	23.8 nmol/L	Positive	Increased recurrent herpes labialis
	[159]	human foreskin fibroblasts infected with CMV AD169	VD_3_, 25(OH)D_3_, and 1,25(OH)_2_D_3_	100 µM	Negative	No effect on CMV replication
**Dengue virus**	[167]	U937-DC-SIGN and THP1 macrophages infected with DENV-2	1,25(OH)_2_D_3_	1 µM	Positive	Inhibition of inflammation caused by dengue virus infection
	[168]	Mouse macrophage infected with DENV-2	VD_3_	0.1 nM	Positive	Inhibition of dengue infection
	[169]	PBMC from 30 VD_3_-fed healthy individuals infected with DENV-2	VD_3_ supplementation	Not measured	Positive	Resistance to dengue infection and inhibition of inflammation
	[170]	Monocyte-derived dendritic cells from 30 VD_3_-fed individuals infected with DENV-2	VD_3_ supplementation	Not measured	Positive	Resistance to dengue infection
	[171]	110 dengue hemorrhagic fever/dengue shock syndrome patients	25(OH)D_3_ deficiency	<50 nmol/L	Positive	Decreased dengue hemorrhagic fever/dengue shock syndrome
**Rotavirus**	[172]	IPEC-J2 cells infected with porcine rotavirus	VD_3_	0.1 µM	Positive	Inhibition of rotavirus replication
	[173]	70 patients	25(OH)D_3_ deficiency	<20 ng/ml	Positive	Increased rotaviral diarrhea
	[174]	IPEC-J2 cells infected with porcine rotavirus	VD_3_	5 µM	Positive	Inhibition of rotavirus infection
	[175]	IPEC-J2 cells or pigs infected with porcine rotavirus	1,25(OH)_2_D_3_	0.1 µM 5000 IU	Positive	Inhibition of rotavirus infection
**PEDV**	[176]	Pigs infected with PEDV	VD_3_ supplementation	115 µg/kg	Positive	Alleviation of intestinal damage by PEDV infection
**HPV**	[177]	2353 sexually active women	25(OH)D_3_ deficiency	<20 ng/ml	Positive	Increased cervicovaginal HPV prevalence
	[178]	67 women with systemic lupus erythematosus	25(OH)D_3_ deficiency	<20 ng/ml	Negative	No effect on HPV infection

The abbreviations used are as follows. DC-SIGN; dendritic cell-specific intercellular adhesion molecule-3-grabbing non-integrin, IPEC; intestinal porcine enterocytes, PEDV; porcine epidemic diarrhea virus.
